# A novel training program for enhancing paramedics’ electrocardiogram interpretation skills: pre–post-evaluation

**DOI:** 10.3389/fmed.2025.1643572

**Published:** 2025-10-06

**Authors:** Keita Iyama, Ryohei Akashi, Makoto Yokoyama, Tomoharu Honda, Kenichiro Maekawa, Komei Miyamoto, Satsuki Tomonaga, Soichi Karatsu, Yu Ikeda, Kensho Baba, Koichi Hayakawa, Osamu Tasaki

**Affiliations:** ^1^Acute and Critical Care Center, Nagasaki University Hospital, Nagasaki, Japan; ^2^Department of Cardiovascular Medicine, Nagasaki University Hospital, Nagasaki, Japan; ^3^Nursing Department, Nagasaki University Hospital, Nagasaki, Japan; ^4^Nagasaki City Fire Department, Nagasaki, Japan; ^5^Nursing Department, Nagasaki Harbor Medical Center, Nagasaki, Japan; ^6^Department of Cardiology, Miyazaki Medical Association Hospital, Miyazaki, Japan; ^7^Emergency and Critical Care Center, Nagasaki Harbor Medical Center, Nagasaki, Japan

**Keywords:** chest pain, education, electrocardiography, emergency medical services, inservice training, training course

## Abstract

**Introduction:**

Early intervention is critical for improving outcomes in patients with acute coronary syndrome (ACS); thus, reducing prehospital duration is essential. Accordingly, 12-lead electrocardiogram (ECG) training is vital for paramedics treating patients with chest pain. However, few studies have reported on such training programs. This study aimed to evaluate the effectiveness of a newly developed 12-lead ECG training program.

**Methods:**

The novel 12-lead ECG training course consisted of six 30-min classroom lectures and practical exercises using eight case scenarios of patients with chest pain. It was conducted for 58 paramedics in Nagasaki Prefecture. Participants completed a 20-question ECG test and a 10-point scale questionnaire before and after the training to assess the course’s impact.

**Results:**

The median number of correct answers on the ECG test significantly improved from 12.5 to 15 after the training (*p* < 0.01). Questionnaire results revealed that anxiety regarding 12-lead ECGs significantly declined from 8.5 to 6 points (*p* < 0.01), whereas confidence significantly increased from 3 to 6 points (*p* < 0.01). The ability to predict ischemic regions using 12-lead ECG increased from 5 to 7 points (*p* < 0.01), and accurate communication of ECG findings to receiving hospitals significantly improved from 5 to 6 points (*p* < 0.01). All participants reported that this training program would benefit their future fieldwork.

**Conclusion:**

Paramedics gained confidence and knowledge through the newly developed 12-lead ECG training program. Continued implementation of this course may help reduce prehospital duration and improve outcomes in patients with ACS in the study region.

## 1 Introduction

Acute coronary syndrome (ACS) prognosis is influenced by the time from onset to treatment, especially in ST-elevation myocardial infarction (STEMI), where prompt and reliable restoration of coronary blood flow without complications is critical. Primary percutaneous coronary intervention (PCI) has become the standard reperfusion therapy, with a required door-to-balloon time (DTBT) of 90 min (1). This 90-min benchmark is the minimum goal, with a target of 60 min when feasible. International guidelines also recommend PCI within 60–90 min of STEMI diagnosis to achieve reperfusion (2). Although shortening DTBT is essential for improving ACS outcomes, studies have shown that reducing DTBT alone does not enhance prognosis (1, 3). Total ischemic time, from symptom onset to reperfusion, is more crucial, and reducing prehospital duration has garnered increasing attention.

The Japan Resuscitation Council (JRC) guidelines recommend prehospital 12-lead ECG recording for patients with suspected ACS, emphasizing the importance of early intervention (4). However, the JRC guidelines also assign a Grade 2D recommendation, with very low evidence, to 12-lead ECG interpretation by non-physician healthcare providers, such as paramedics and nurses. In Japan, there are basically no emergency physicians outside hospitals, and paramedics are the only medical professionals who come into contact with patients in prehospital settings. However, they are not authorized to treat patients with ACS; rather, their duty is to transport patients to a hospital capable of performing emergency cardiac catheterization if ACS is suspected from ECG findings or physical examination or to a general emergency hospital if it is not suspected. Treatment is then initiated by a physician upon arrival at the hospital. Although paramedics play a key role in prehospital care, past surveys show that such healthcare providers lack sufficient knowledge and confidence in interpreting 12-lead ECGs (5). Conversely, advances in technology have enabled some regions to transmit prehospital 12-lead ECGs to physicians for direct interpretation via the web (6). In such areas, paramedic interpretation is less critical, but most regions lack ECG transmission systems owing to economic constraints. Therefore, many areas in Japan still depend on paramedics for prehospital 12-lead ECG interpretation. As paramedics in Japan do not receive routine training or have mandatory responsibilities regarding ECG, learning how to interpret ECG results is largely left to individual initiative.

To improve the quality of 12-lead ECG interpretation by paramedics, appropriate training is necessary. However, no standardized programs currently exist, and establishing regional educational initiatives remains an important goal (4). As a preliminary effort, we surveyed 395 paramedics across Nagasaki Prefecture to assess 12-lead ECG awareness (5). The results showed that although paramedics understood that their 12-lead ECG interpretation skills impact ACS outcomes, they lacked confidence and had insufficient training (5). Ideally, awareness of their influence on prognosis would motivate adequate self-study, but limitations such as self-learning constraints and poor educational environments were apparent. Given the high demand for 12-lead ECG training, we designed a specialized course for paramedics in prehospital settings.

Previous research has revealed that lectures and workshops are more effective for teaching electrocardiograms than self-study (7, 8). Therefore, based on preliminary survey findings, we identified educational needs and developed a 12-lead ECG training program tailored to active paramedics, which we then began offering regionally (9). The survey revealed that the majority of paramedics preferred practical learning over classroom lectures for ACS response training. Accordingly, our course included interpreting 12-lead ECGs, selecting appropriate medical facilities, and communicating effectively with hospitals.

In this study, all data were anonymized to ensure privacy. This study was originally published in Japanese language in the Journal of the Japanese Association for Acute Medicine (10). In this study, we report on the training program we developed to enhance paramedics’ 12-lead ECG interpretation skills, aiming to improve prognosis outcomes in patients with ACS by reducing transport time to hospital and expediting emergency cardiac catheterization activation.

## 2 Materials and methods

The training course consisted of six 30-min classroom lectures and practical exercises with eight case scenarios using a low-fidelity simulation. The course involved no pre-learning, and it was a face-to-face course and not an online lecture. The original textbook was provided to the participants. During the case scenarios, participants conducted patient interviews, performed physical examinations, and performed 12-lead ECGs, as well as selected transport destination medical facilities and negotiated care for a simulated patient with chest pain (Table 1). Supplementary Table 1 describes eight case scenarios. The instructors of this course were cardiovascular physicians, emergency physicians, emergency nurses, and paramedics who were certified at level 2 or higher by the Japan Heart Rhythm Society’s ECG, and there were at least three instructors for each case scenario. All participants experienced all eight scenarios and had a debriefing by instructors.

**Table 1:** Training course timetable.

TIME		Lecture Topic
10:00–10:30	0:30	Guidance/pretest/opening ceremony
10:30–11:00	0:30	Lecture 1: Basics of 12-lead ECG
11:00–11:30	0:30	Lecture 2: General theory of arrhythmia
11:30–11:40	0:10	Break
11:40–12:10	0:30	Lecture 3: Estimation of the ischemic area
12:10–12:40	0:30	Lecture 4: Various ECGs of acute coronary syndrome
12:40–13:30	0:50	Lunch
13:30–14:00	0:30	Lecture 5: Physical findings
14:00–14:30	0:30	Lecture 6: Required initial response
14:30–14:45	0:15	Break
14:45–14:55	0:10	Guidance: Practical training
14:55–15:00	0:05	Break
15:00–17:10	2:10	Practical training: 8 Case scenarios
17:10–17:15	0:05	Break
17:15–17:45	0:30	Post-test/closing ceremony

ECG, electrocardiogram.

We implemented the 12-lead ECG training program developed for paramedics from fire departments in Nagasaki Prefecture. An announcement regarding a 12-lead ECG training program was made by the fire department. All 58 paramedics who voluntarily expressed an intention to participate in this program were selected as research participants. Previous studies suggest that mock tests are effective for evaluating 12-lead ECG interpretation skills (11, 12). Therefore, the primary endpoints of this study were the scores on a 20-question written test—created with reference to the Japan Heart Rhythm Society’s Level 3 ECG Certification examination question bank—and participants’ confidence in interpreting 12-lead ECGs, measured via questionnaire surveys. Pretests and pre-questionnaires were conducted before training, and post-tests and post-questionnaires were administered afterward. Questionnaire content was created based on the previous research and is provided in Table 2 (5). The questionnaire used a 10-point Likert scale, ranging from “strongly disagree” to “strongly agree.” Participants rated their agreement with each item on a scale from 1 to 10, where 1 = strongly disagree, 5 = somewhat disagree, 6 = somewhat agree, and 10 = strongly agree. A median score of ≥6 was interpreted as agreement, whereas a score of ≤5 indicated disagreement.

**Table 2:** Results of questionnaire survey.

The participants assigned a score (1–10) to each question (1 = strongly disagree, 10 = strongly agree). (*n* = 58)	Precourse		Postcourse		*p*-value
	Median (IQR)	Percentage of respondents scoring 6 or more points. (%)	Median (IQR)	Percentage of respondents scoring 6 or more points. (%)	
Q1. I am confident in my basic knowledge of 12-lead ECG.	3 (2–4.25)	7	6 (5–7)	59	***<0.01***
Q2. I am confident in my ability to read 12-lead ECG.	3 (1–5)	9	6 (5–7)	59	***<0.01***
Q3. I am anxious in my ability to read 12-lead ECG.	8.5 (7–10)	81	6 (5–7)	62	***<0.01***
Q4. I have learned enough about 12-lead ECG.	3 (1.75–4)	4	5 (3–6)	27	***<0.01***
Q5. I would like to actively engage in self-study about 12-lead ECG.	10 (8–10)	96	10 (9.75–10)	100	***<0.01***
Q6. Reading ability of 12-lead ECG is a necessary basic skill for paramedics.	10 (8–10)	95	10 (9–10)	100	***0.01***
Q7. I can predict ischemic areas and urgency through the 12-lead ECG.	5 (3–7)	37	7 (6–8.25)	82	***<0.01***
Q8. I can accurately convey information on the 12-lead ECG results to the hospital.	5 (3–5)	18	6 (5–8)	71	***<0.01***
Q9. Self-study about 12-lead ECG has limitations.	8 (7–10)	86	8 (7–10)	85	0.60
Q10. Paramedics’ ability to read 12-lead ECG affects the prognosis of patients with ACS.	8 (7–10)	90	10 (8–10)	96	***<0.01***
Q11. Paramedics should be well-learned about ACS response.	10 (8–10)	97	10 (9–10)	97	0.06
Q12. Paramedics should be routinely educated and trained to prepare for ACS management.	10 (8–10)	98	10 (8–10)	98	0.13
Q13. Education and training to read 12-lead ECG are essential.	10 (8–10)	98	10 (9–10)	100	***0.03***
Q14. The pathology from 12-lead ECG alone is difficult to ascertain.	8 (7–9.25)	86	9 (8–10)	95	***<0.01***
Q15. Performing 12-lead ECG in an on-site field is difficult.	4 (3–6)	28	3 (1–5)	17	***0.03***
Q16. Today’s training course will be useful for future activities.	ー	ー	10 (10–10)	100	ー
Number of correct answers to 20 test questions.	12.5 (11–15)	ー	15 (13.75–17)	ー	***<0.01***

*p*-values in bold italics indicate a statistically significant difference between pre-training and post-training results. ECG, electrocardiogram; IQR, interquartile range.

Median scores for the questionnaire and ECG test were calculated, and the Wilcoxon signed-rank test was used to assess the training course’s effectiveness and its influence on paramedics’ awareness of 12-lead ECGs. Statistical analysis was performed using JMP Pro 18 (SAS Institute Inc.), with a significance level of 0.05. This study was reported in accordance with the Strengthening the Reporting of Observational Studies in Epidemiology (STROBE) guidelines for cross-sectional studies.

### 2.1 Ethics statement

The study conformed to the principles of the 1975 Declaration of Helsinki and its later revisions and obtained approval from the Ethics Committee of Nagasaki University (approval number: 20103001–5). Informed consent was obtained from all study participants. Additionally, no animal studies were used.

## 3 Results

The background of the 58 participating paramedics is shown in Table 3. Questions with a median score of ≥6 before training (indicating agreement) included Q3 (anxiety), Q5 (self-improvement), Q6 (recognition of importance), Q9 (limitation of self-study), Q10 (own ability affecting prognosis), Q11 (should study hard), Q12 (should train hard), Q13 (necessity of education and training), and Q14 (difficult to understand pathology) (Table 2). During pretraining, participants lacked confidence in 12-lead ECGs and felt anxious (Q1–3), considered their learning insufficient (Q4), and wanted to self-improve (Q5). They also recognized 12-lead ECG interpretation as a basic skill (Q6), but felt unable to make judgments from the waveforms or accurately communicate results to transport destinations (Q7 and Q8) while also noting limitations in 12-lead ECG-related self-learning (Q9). Paramedics believed that their 12-lead ECG interpretation ability affected prognosis (Q10), had high learning standards (Q11 and Q12), and understood its importance (Q13). However, they acknowledged that 12-lead ECGs alone could not detect all conditions (Q14) and reported no major difficulties in implementing them in prehospital settings (Q15). The median number of correct answers on the 20-question ECG test before training was 12.5. When grouped by past ECG training, those with training experience were more confident in their ECG skills (Q2) and responded that they could convey information appropriately (Q8). After the training course, the group with experience reported greater motivation to self-study (Q5) and had higher ECG test scores than the group without experience (Table 4).

**Table 3:** Participant characteristics.

Age (median, IQR)		34 (28–38)
Sex (*n*, %)	Male	56 (96.6%)
	Female	2 (3.4%)
Practical experience (*n*, %)	<5y	17 (29.3%)
	≧5y, <10y	20 (34.5%)
	≧10y, <15y	12 (20.7%)
	≧15y, <20y	8 (13.8%)
	≧20y, <25y	0 (0%)
	≧25y	1 (1.7%)
Other ECG-related training courses attended (*n*, %)	Yes	22 (37.9%)
	No	36 (62.1%)

ECG, electrocardiogram; IQR, interquartile range.

**Table 4:** Results by whether or not with experience in an ECG-related training course.

The participants assigned a score (1–10) to each question (1 = strongly disagree, 10 = strongly agree). (*n* = 58)	Precourse			Postcourse		
	Experience with other ECG-related training courses. (*n* = 22)	No experience with ECG-related training courses. (*n* = 36)	*p*-value	Experience with other ECG-related training courses. (*n* = 22)	No experience with ECG-related training courses. (*n* = 36)	*p*-value
Q1. I am confident in my basic knowledge of 12-lead ECG.	3.5 (2–5)	3 (1.25–3.75)	0.08	6 (5.75–8)	5.5 (4.25–7)	0.05
Q2. I am confident in my ability to read a 12-lead ECG.	5 (1–5)	3 (1–3)	***0.05***	7 (5–8)	5.5 (4–6)	***0.02***
Q3. I am anxious about my ability to read a 12-lead ECG.	8 (5–10)	9 (8–10)	0.20	5 (4–7)	6 (5–8)	0.08
Q4. I have learned enough about 12-lead ECG.	3 (1–4.25)	3 (2–3.75)	0.99	5 (2.75–6)	4.5 (3–6)	0.79
Q5. I would like to actively engage in self-study about 12-lead ECG.	9.5 (8–10)	10 (8–10)	0.71	10 (10–10)	8.25 (10–10)	***0.03***
Q6.Reading the ability of a 12-lead ECG is a necessary basic skill for paramedics.	10 (8–10)	9.5 (8–10)	0.85	10 (9.75–10)	10 (8–10)	0.19
Q7. I can predict ischemic areas and urgency through the 12-lead ECG.	5 (3–7.25)	5 (2.25–7)	0.36	7.5 (6–9)	7 (6–8)	0.16
Q8. I can accurately convey information on the 12-lead ECG results to the hospital.	5 (5–5.25)	3 (2–5)	***0.01***	7 (6–8.25)	6 (5–7)	***0.01***
Q9. Self-study about the 12-lead ECG has limitations.	8 (6–10)	8 (7–10)	0.77	8 (6.75–10)	9 (7–10)	0.36
Q10. Paramedics’ ability to read 12-lead ECG affects the prognosis of patients with ACS.	9 (7.75–10)	8 (6.25–10)	0.23	10 (9–10)	9.5 (8–10)	0.08
Q11. Paramedics should be well learned in ACS response.	10 (8–10)	10 (8–10)	0.84	10 (9–10)	10 (8.25–10)	0.33
Q12. Paramedics should be routinely educated and trained to prepare for ACS management.	10 (7.75–10)	10 (8–10)	0.93	10 (8–10)	10 (8–10)	0.38
Q13. Education and training to read a 12-lead ECG are essential.	10 (8–10)	10 (8–10)	0.57	10 (9–10)	10 (8.25–10)	0.42
Q14. The pathology on a 12-lead ECG alone is difficult to ascertain.	8 (7–10)	8 (6.25–8)	0.25	10 (7.75–10)	9 (8–10)	0.63
Q15. Performing a 12-lead ECG in the on-site field is difficult.	4 (1.75–7)	4 (3–5)	0.80	3 (1–4)	3 (1.25–5)	0.62
Q16. Today’s training course will be useful for future activities.	ー	ー	ー	10 (10–10)	10 (10–10)	0.21
Number of correct answers to 20 test questions.	14.5 (11.75–15)	12 (11–14)	0.09	16.5 (15–18)	15 (12.25–16.75)	***0.04***

*p*-values in bold italics indicate a statistically significant difference between pretraining and post-training results. ECG, electrocardiogram; IQR, interquartile range.

Significant differences in questionnaire results before and after training were found in 12 questions: 1–8, 10, and 13–15 (Table 2). For the primary endpoints, questions 1 (“confidence in basic knowledge”) and 2 (“confidence in interpretation”) showed significant increases from a median score of 3 to 6 (*p* < 0.01, respectively), and the percentage of participants answering with ≥6 increased from 7 and 9% to 59 and 59%, respectively. This indicated a marked increase in confident participants post-training. In contrast, question 3 (“anxiety about interpretation”) significantly decreased from a median score of 8.5 to 6 after training (*p* < 0.01), but 62% of participants still reported anxiety, with scores of ≥6. For questions 7 (“predicting ischemic regions and judging urgency”) and 8 (“ability to accurately communicate 12-lead ECG interpretation results to the transport destination”), the scores significantly increased after training (5 vs. 7, 5 vs. 6, *p* < 0.01, respectively), with the percentage of participants responding with ≥6 increasing to 82 and 71%, respectively.

For the other primary endpoint, the ECG test, the median number of correct answers increased significantly from 12.5 to 15 after training (*p* < 0.01). Additionally, for post-training question 16 (“today’s training course will be useful for future activities”), 100% of participants responded with ≥6.

## 4 Discussion

This study aimed to improve the prognosis of patients with ACS by enhancing the quality of prehospital 12-lead ECG interpretation by paramedics. In the United States, the national standard curriculum for paramedics includes a basic understanding of the pathophysiology of acute myocardial infarction and electrocardiogram findings. However, such a standard does not yet exist in Japan (13). A previous preliminary survey in Japan revealed that, although paramedics recognized the impact of their 12-lead ECG interpretation skills on ACS prognosis, they lacked confidence and had insufficient knowledge (5). Ideally, understanding the influence of their skills on patient outcomes should motivate them to study and gain confidence; however, limitations in self-learning and inadequate learning environments restrict their development. Due to the high demand for 12-lead ECG training courses, we established a training program tailored for paramedics in prehospital settings. The training course, based on feedback from preliminary research, improved the knowledge and confidence of participants. Notably, course graduates could potentially reduce the prehospital duration and ensure appropriate transport during prehospital care.

Regarding basic 12-lead ECG interpretation, significant improvements in ECG test scores after the training suggest knowledge acquisition by participants. However, knowledge alone is insufficient for field implementation; practical opportunities are essential (14, 15). Previous studies have shown that lecture-style ECG education supplemented with small-group practical training in ECG interpretation is effective (16, 17). Therefore, this training program also consisted of two parts: lectures and practical training. The developed training program included theoretical input via classroom lectures and practical output through case scenarios. In the practical training, participants conducted physical examinations, obtained medical histories, selected transport destination medical facilities, and negotiated with medical facilities for patient acceptance. Therefore, it is clinically significant that 71% of participants reported being able to accurately communicate 12-lead ECG interpretation results to transport destinations (Q8). Importantly, effective communication ensures timely intervention by physicians and nurses, thereby reducing ischemic time. Additionally, 82% of participants could predict ischemic regions and assess urgency from 12-lead ECG findings (Q7), which is crucial for early transport.

Although confidence in basic knowledge (Q1) and ECG interpretation (Q2) significantly increased after training and anxiety about interpretation (Q3) significantly decreased, 62% of participants still reported anxiety post-training. Although the median score decreased from 8.5 to 6 (*p* < 0.01), indicating that the training reduced anxiety, a single session could not fully eliminate it. Similarly, although the learning status (Q4) significantly improved after training, only 27% of participants felt that their learning was sufficient, again suggesting that one session was insufficient. On the other hand, among participants in the group with experience in other ECG-related training courses, there was a significant increase in ECG scores after completing our course compared to participants in the group without experience, and their motivation for self-study was also higher. This finding suggests that repeated training courses are effective. The Fire and Disaster Management Agency advocates ongoing on-the-job training as part of continuous education in emergency medical services. Repeated participation in training, real-world application, and feedback from physicians could further reduce anxiety and reinforce learning retention (18). Kolb proposed that humans learn through experience and described the learning process as a cycle comprising four stages: concrete experience, reflective observation, abstract conceptualization, and active experimentation (19). Participants in this study were paramedics who regularly engaged in field activities and were thus considered to have extensive concrete experience and reflective observation. This training program has the potential to stimulate their abstract conceptualization and active experimentation, effectively promoting the cycle of experiential learning, leading to improved lifelong learning and self-learning.

During pretraining, participants were already motivated for self-improvement (Q5), and after training, all participants scored ≥6; thus, participating in the training further improved motivation. Question 10 (“one’s 12-lead ECG interpretation ability affects patient prognosis”) showed significantly increased awareness of prehospital intervention in ACS response. Participants also understood that 12-lead ECGs are not the only diagnostic tool for chest pain and that medical history and physical findings are also crucial (Q14).

The training course included six 30-min lectures and eight case scenarios, each lasting 15 min. The majority of participants felt that the time allocation was appropriate. In the study region, the start time was set at 10:00 to accommodate participants finishing night shifts, although it can be adjusted based on local needs. We are also considering converting the classroom portion to e-learning in the future.

This study has some limitations. First, it did not investigate whether training directly affected total ischemic time or patient prognosis. Second, selection bias in the originally motivated population is possible, given that paramedics attending the training program are targeted. As this study adopted a single-arm pre–post comparison design without a control group, it is difficult to confirm generalizability. Third, since the content of the ECG tests conducted before and after training was very similar, it may be difficult to evaluate post-training increases in test scores using simple criteria. Fourth, we did not conduct a long-term follow-up to assess paramedics’ knowledge retention and clinical impact. Additionally, the findings are limited to the study region. Fifth, we did not compare the effectiveness of this training course with that of other training courses, such as online lectures or those using different textbooks.

## 5 Conclusion

In this study, we developed and implemented a 12-lead ECG training program for paramedics to improve the prognosis of patients with ACS. The training successfully increased participants’ confidence and knowledge in interpreting 12-lead ECGs. After attending the training program, paramedics are expected to confidently and accurately read ECGs and perform smooth field activities, which will contribute to reduced ischemic time and improved outcomes in patients with chest pain in the future. We plan to continue this training program nationwide, aiming to improve the prognosis of patients with ACS.

## Data Availability

The original contributions presented in the study are included in the article https://doi.org/10.1002/jja2.13000, further inquiries can be directed to the corresponding author.

## Author’s note

This is a English language translation of “救急救命士の12誘導心電図判読能力向上に向けたトレーニングプログラム (A novel training program to improve paramedics’ 12–lead electrocardiogram interpretation skills)” originally published in Journal of Japanese Association for Acute Medicine (JJAAM). Permission was granted by Mikio Nakajima, the editor in chief of JJAAM.
