# Supplementary material for: A novel training program for enhancing paramedics’ electrocardiogram interpretation skills: pre–post-evaluation
Source: Front Med (Lausanne). 2025 Oct 6;12:1643572. doi: 10.3389/fmed.2025.1643572 (PMC12535971; doi:10.3389/fmed.2025.1643572)
Supplement: Supplementary file 1 [file Table_1.docx]

Supplementary Material

**Supplementary Table 1.** Case description of the eight case scenarios.

| **Case No.** | **Case description.** |
| --- | --- |
| Case 1 | Simple STEMI (anterior wall) |
| Case 2 | Acute heart failure (Clinical Scenario 1) |
| Case 3 | Acute pulmonary thromboembolism |
| Case 4 | Acute aortic dissection |
| Case 5 | Aortic valve stenosis |
| Case 6 | Inferior wall infarction + bradycardia (AV block) |
| Case 7 | Inferior wall infarction + papillary muscle rupture + acute mitral regurgitation |
| Case 8 | Non-cardiac case with chest pain as the chief complaint (normal ECG) |

STEMI, ST-elevation myocardial infarction; AV block, atrioventricular block; ECG, electrocardiogram
